# Do Longer Intervals between Challenges Reduce the Risk of Adverse Reactions in Oral Wheat Challenges?

**DOI:** 10.1371/journal.pone.0143717

**Published:** 2015-12-01

**Authors:** Noriyuki Yanagida, Takanori Imai, Sakura Sato, Motohiro Ebisawa

**Affiliations:** 1 Department of Pediatrics, Sagamihara National Hospital, Kanagawa, Japan; 2 Clinical Research Center for Allergy and Rheumatology, Sagamihara National Hospital, Kanagawa, Japan; Cincinnati Children's Hospital Medical Center, University of Cincinnati College of Medicine, UNITED STATES

## Abstract

**Background:**

The use of oral food challenges (OFCs) in clinics is limited because they are complicated and associated with anaphylactic symptoms. To increase their use, it is necessary to develop novel, effective, and safe methods. However, the effectiveness of different OFCs has not been compared.

**Objective:**

To investigate the effect of ingestion methods on wheat allergy symptoms and treatment during OFCs.

**Method:**

Without changing the total challenge dose, we changed the administration method from a 5-installment dose titration every 15 min (15-min interval method) to 3 installments every 30 min (30-min interval method). We retrospectively reviewed and compared the results of 65 positive 15-min interval wheat challenge tests conducted between July 2005 and February 2008 and 87 positive 30-min interval tests conducted between March 2008 and December 2009.

**Results:**

A history of immediate symptoms was more common for the 30-min interval method; however, no difference between methods was observed in other background parameters. Switching from the 15-min to the 30-min interval method did not increase symptoms or require treatment. The rate of cardiovascular symptoms (*p* = 0.032), and adrenaline use (*p* = 0.017) was significantly lower with the 30-min interval method. The results did not change after adjusting for the effects of immediate symptom history in multivariate analysis.

**Conclusion:**

This study suggests that the 30-min interval method reduces the risk of adverse events, compared to the 15-min interval method.

## Introduction

Oral food challenges (OFC) are performed to diagnose and confirm food allergies, and to evaluate food tolerance [1–6]. However, conducting OFCs is complicated, and the development of anaphylactic symptoms can risk patient health. Thus, their use is limited in clinics. To increase the use of OFCs, the development of novel, effective, and safe methods is needed. When only subjective symptoms are present in older children, a double-blind test is necessary. However, an open challenge test is considered adequate for children aged 3 years and younger, who account for the majority of cases [7].

To date, the effectiveness of different OFC methods has not been compared. Thus, we retrospectively compared results from two OFCs conducted in our hospital to develop an improved OFC methodology.

## Methods

### Inclusion and exclusion criteria

OFCs were conducted to verify the diagnosis and tolerance of food allergies. Patients with suspected wheat allergies, including those who experienced an allergic reaction to wheat that improved when wheat was eliminated, and who were positive for wheat-specific IgE, were also enrolled. We included two types of patients: those with a clinical history of allergic reaction in close relation to wheat ingestion and those with deteriorating symptoms, such as eczema, which could be due to wheat. Anaphylaxis was defined as described by the World Allergy Organization (WAO) [8, 9]. A history of anaphylaxis was not established as an exclusion criterion, and no exclusion criteria were established with respect to wheat-specific IgE antibody titers. We did not use the skin prick test [10]. We used total and wheat-specific IgE values (Immuno CAPTM; Thermo Fisher Scientific, United States) measured within six months of the OFC.

OFCs were not performed on patients with symptoms, such as eczema or respiratory infections, which would affect the determination of OFC results. The OFC was conducted following removal of drugs that could affect the results, including antihistamines. Patients with a history of immediate reaction to wheat within the past 6 months were also excluded.

### Study entry

Of 406 wheat OFCs conducted in the pediatric unit of Sagamihara National Hospital from July 2005 to December 2009, positive symptoms were induced in 187 cases. In cases where symptoms were induced upon intake at home, but no or very mild symptoms considered to have no relation to food induced symptoms were observed during the test, the patients were considered positive. We excluded 17 such patients from the study. In patients who underwent OFCs with the same allergen more than once, the initial OFC was used for analysis. We excluded 18 patients who underwent the same OFC.

Among 152 cases, 65 positive cases were retrospectively reviewed using the 15-min interval method from July 2005 to February 2008, and 87 positive cases were obtained using the 30-min interval method from March 2008 to December 2009.

### OFC testing method

The OFC was evaluated using an open method with either a 15-min or a 30-min interval. In the 15-min interval method, five doses were administered, starting from 1/16 of the total load, followed by 1/16, 1/8, 1/4, and 1/2 every 15 min, up to 60 min. In the 30-min interval method, three doses were administered, starting from 1/8 of the total load, followed by 3/8 and 1/2 for 30 min, up to 60 min.

The OFC test was conducted using Japanese wheat noodles (50 g) containing 130 mg wheat protein, prepared in the nutrient control room of our hospital. In the 15-min interval method, 3 g (78 mg), 3 g (78 mg), 6 g (156 mg), 12 g (312 mg), and 26 g (676 mg) of Japanese wheat noodles were used, whereas in the 30-min interval method, 6 g (156 mg), 18 g (234 mg), and 26 g (676 mg) were used. We observed the patients for at least 3 h after final administration.

### Positive OFC criteria

Positive OFCs were determined based on the presence of induced symptoms (Table 1). We assessed symptoms using Table 1, which was a modified from a grading system developed by Sampson *et al*. [11]. Obvious symptoms falling within grades 2 and 3 (Table 1) were considered positive criteria, including a range of skin symptoms (urticaria, erythema, and pruritus), respiratory tract symptoms (hoarseness, sore throat, dysphagia, cough and wheezing), and gastrointestinal symptoms (vomiting and diarrhea).

**Table 1:** Grading of symptoms.

	1 (mild)	2 (moderate)	3 (severe)
Skin	Localized urticarial or exanthema or wheal or pruritis	Generalized urticarial or exanthema or wheal or pruritis	-
	Swollen eyelid or lip	Swollen face	-
Gastrointestinal tract	Pruritus of the throat or oral cavity	Throat pain	Throat tightness, difficulty swallowing
	Mild abdominal pain	Moderate abdominal pain	Cramps
	Nausea, emesis, diarrhea	Recurrent emesis or diarrhea	Continuous emesis, loss of bowel control
Respiratory tract	Intermittent cough, nasal congestion, sneezing, rhinorrhea	Repetitive cough	Persistent cough, hoarseness, “barky” cough
	-	Chest tightness, mild wheezing	Apparent wheezing, dyspnea, cyanosis, saturation <92%, swallowing or speaking difficulties, throat tightness, respiratory arrest
Cardiovascular	-	Pale face, mild hypotension, tachycardia (increase >15 beats/min)	Hypotension, dysrhythmia, severe bradycardia, cardiac arrest
Neurological	Change in activity level, tiredness	“Light-headedness,” feeling of “pending doom,” somnolence	Confusion, loss of consciousness, incontinence

The severity score should be based on the organ system most affected.

Hypotension was defined as systolic blood pressure of <70 mmHg (ages, 1 month to 1 year), <(70 mmHg + [2 × age]) (ages, 1–10 years), and <90 mmHg (>11 years).

Mild hypotension was defined as systolic blood pressure of <80 mmHg (ages, 1 month to 1 year), <(80 mmHg + [2 × age]) (ages, 1–10 years), and <100 mmHg (>11 years).

Total severity scores were defined as the grade of cardiovascular symptoms + the grade of respiratory symptoms + the maximum grade of other symptoms.

Symptom severity was assessed on the basis of a total severity score. Total severity scores were defined as the sum of the scores for the five organ systems, as described in Table 1.

### Treatment of induced symptoms

Appropriate measures, including fluid resuscitation, oxygenation, antihistamine and steroids administration, β_2_ stimulant inhalation, and adrenaline injections, were applied based on symptom severity. Intramuscular adrenaline injections were used for strong gastrointestinal symptoms, airway obstruction, hypotension, loss of consciousness, tightness of throat, and in cases respiratory symptoms persisted after β_2_ stimulant inhalation.

### Statistical analyses

The results are expressed as median value and 25–75th percentiles. For statistical comparisons between 2 groups, we used the Mann-Whitney U test, Fisher’s exact test, or multiple logistic regression for multivariate analysis. A *p*-value less than 0.05 was considered statistically significant. The data were statistically analyzed using SPSS 20.0 (IBM Corporation, Armonk, NY, USA).

### Ethical considerations

The possibility of symptoms was explained orally and in writing to subject guardians, and written consent was obtained. This study was conducted according to the principles of the Declaration of Helsinki and the guidelines on clinical research from the Ministry of Health, Labor, and Welfare, and was carried out after obtaining approval from the Ethic Committee of Sagamihara National Hospital (approval number 18).

## Results

### Comparison of patient clinical backgrounds

The clinical backgrounds of patients undergoing the 15-min and 30-min interval methods were compared (Table 2). A clinical history of immediate-type symptoms was found in 60% of the patients in the 15-min interval group and 85% of the patients in the 30-min interval group (*p* = 0.001). There was no difference in clinical history of anaphylactic symptoms, wheat-specific IgE, or age.

**Table 2:** Comparison of challenge-positive patient profiles.

Caracteristics	15 min interval(n = 65)	30 min interval(n = 87)	*p*-value
Gender (male)	42 (65%)	58 (67%)	0.463
Age (years)	2.6 (1.9–4.3)	3.8 (2.0–5.0)	0.065
History of immediate reaction to wheat	39 (60%)	74 (85%)	0.001
History of anaphylactic reaction to wheat	8 (12%)	22 (25%)	0.063
Atopic dermatitis	37 (55%)	45 (51%)	0.628
Asthma	11 (16%)	19 (21%)	0.539
Total IgE (IU/mL)	267 (140–1020)	356 (119–743)	0.749
Wheat-specific IgE (kUA/L)	4.6 (1.9–22.9)	8.4 (2.0–9.7)	0.447

Age, total IgE, and wheat-specific IgE are expressed as the median (25–75th percentile).

Comparisons were made with Fisher’s exact test or Mann—Whitney U test.

*p* < 0.05 was considered statistically significant.

### Review of OFC results

#### Total loading

**Fig 1:**
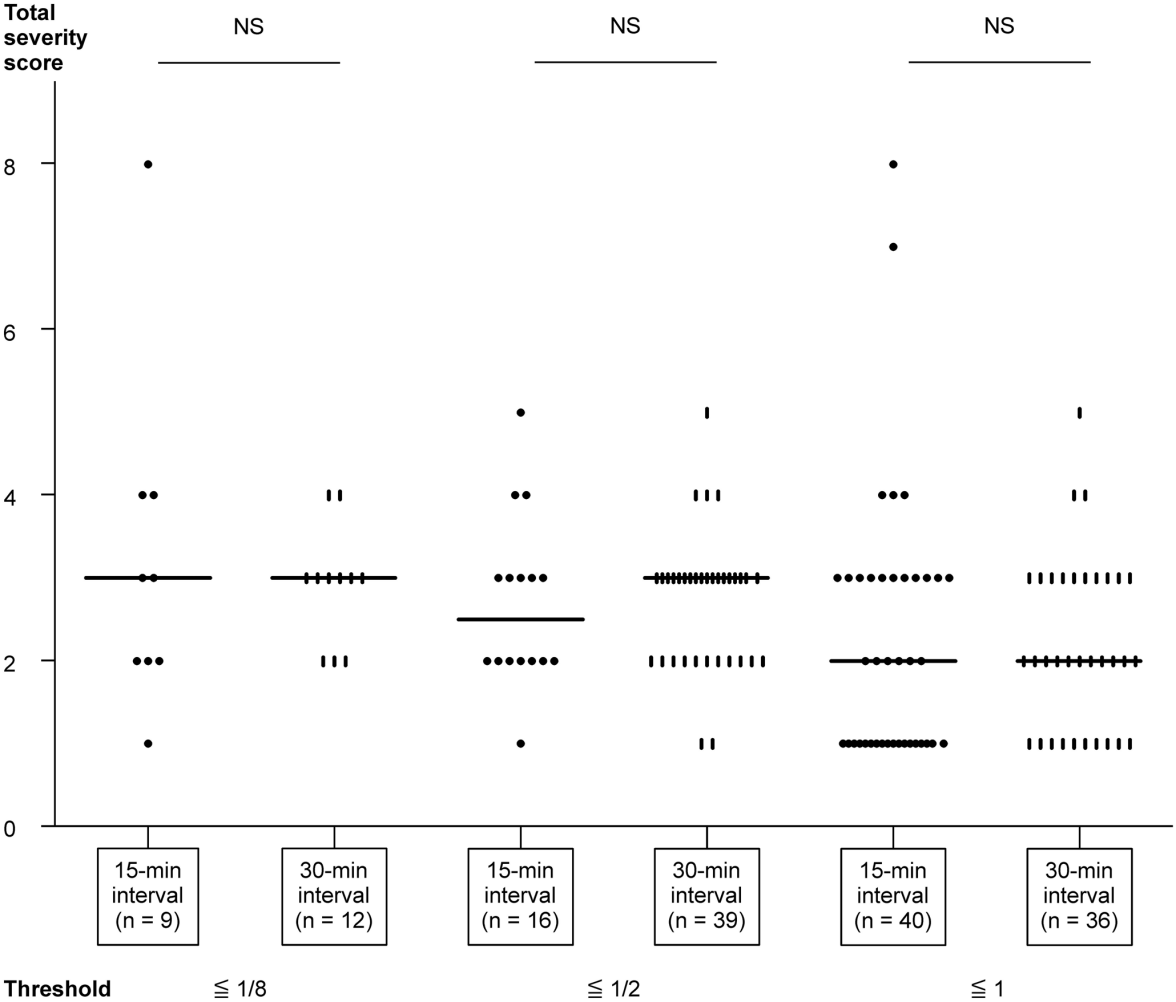
Threshold of oral food challenge test and total severity score. Total severity scores were defined as described in Table 1. The horizontal line represents the median.

The total loading amounts in positive patients (positive threshold) and total severity scores were compared using two methods (Fig 1). A threshold of 1/8 or lower was observed in 8% of the patients in the 15-min interval group and in 12% of the patients in the 30-min interval group. Thresholds between 1/8 and 1/2 accounted for 20% and 35% of the patients in the 15-min and 30-min interval groups, respectively. Finally, thresholds greater than 1/2 were observed in 72% and 53% of the patients in the 15-min and 30-min interval groups, respectively. The median total severity score in the 30-min and 15-min interval groups were 2.0. The total severity score of the 30-min interval group did not differ from that of the 15-min interval group at any threshold. Nevertheless, among patients in the 15-min interval, 1 patient had a total severity score of 7 and 2 patients had a score of 8, whereas the total severity scores did not exceed 6 when the 30-min interval method was used.

#### Induced symptoms

The rate of cardiovascular symptoms was significantly higher for the 15-min interval method (6%) than for the 30-min interval method (0%; *p* = 0.032; Table 3). All cardiac symptoms involved hypovolemic shock. No differences were observed for other symptoms between the methods. In addition, no neurological symptoms were observed.

**Table 3:** Induced reactions and treatments for positive challenges.

	15 min interval(n = 65)	30 min interval(n = 87)	*p*-value
Symptoms			
Skin	54 (83%)	72 (83%)	1.000
Respiratory	44 (68%)	58 (67%)	1.000
Gastrointestinal	6 (9%)	11 (13%)	0.608
Cardiovascular	4 (6%)	0 (0%)	0.032
Neurological	0 (0%)	0 (0%)	1.000
Multiple-organ symptoms	35 (54%)	54 (62%)	0.323
Treatment			
Antihistamines	26 (40%)	49 (56%)	0.051
β_2_ stimulant inhalation	36 (55%)	44 (51%)	0.623
Steroids	21 (32%)	22 (25%)	0.367
Adrenaline	9 (14%)	3 (3%)	0.030
Any treatment	44 (68%)	69 (79%)	0.133

p < 0.05 was considered statistically significant.

#### Treatment of induced symptoms

The use of adrenaline was significantly higher for the 15-min interval method (14%) than for the 30-min interval method (3%; *p* = 0.032; Table 3). No other differences were observed in treatments between methods.

S1 Table describes the 12 cases that required adrenaline. All cases had a combination of respiratory and skin symptoms. In the 15-min interval method, four cases had cardiac symptoms. In the 30-min interval method, adrenaline was given to patients with respiratory tract symptoms.

#### Multivariate analysis

A multivariate analysis was conducted to examine circulatory organ symptoms, adrenaline administration, and differences between the methods. After adjusting the effects in the 15-min interval method for the presence or absence of immediate symptoms, where a difference was observed in the target background and the wheat specific IgE value had clinical significance in the severity evaluation [12], it was found that this method had significantly higher cardiovascular symptoms (*p* = 0.015). Adrenaline administration occurred more frequently in the 15-min interval group (*p* = 0.045).

## Discussion

The OFC test is the gold standard for food allergy diagnosis [1, 2]. The PRACTALL consensus report [13], an international paper discussing the best practices for OFC, recommended an interval of at least 20 min or more between challenges; however, different methods have never been compared. In this study, we compared two types of OFCs with different intake intervals to identify a safer and more efficient technique.

### Comparison of induced symptoms and treatment

When subjects with positive OFCs using different methods were retrospectively compared, a difference was observed only in the immediate clinical history. Because OFCs are generally conducted using the 30-min method in cases with a high risk of immediate allergic reactions, patients may experience more adverse effects.

While the initial load was 1/16 in the 15-min interval method, it increased to 1/8 in the 30-min method. Nevertheless, some cases showed positive symptoms at this load, although no cases required adrenaline administration. Similarly, Rolinck-Werninghaus *et al*. reported that severe symptoms more commonly occurred at higher wheat loads than lower loads [14].

In contrast, symptoms were not observed until the final intake in most patients using the 30-min interval method. Therefore, the administration could be altered by extending the interval time to 30 min or longer or increasing the initial load to more than 1/8.

Compared to the 15-min interval method, the 30-min interval method was not associated with an increase in severe symptoms, treatment requirement, cardiovascular symptoms, or adrenaline treatment. This is because lengthening the observation period enabled the identification of symptoms using a smaller load, resulting in decreased severity. Nevertheless, the reason remains unclear.

### Appropriate interval for OFCs

There are many restrictions associated with the 15-min interval method compared with the 30-min method. Young patients, who account for many OFC subjects, can become stressed by the frequent intake of OFC test meals when using 15-min intervals. In the 30-min interval method, the time from initial intake to final intake was 1 h, which is the same as that of the conventional 15-min interval method; however, the procedure is simpler. Further, because there are fewer administrations (3 versus 5), the 30-min method is simpler and has a lower risk of incorrect administration. The time to maximum urine concentration of urinary metabolites of wheat is said to range from the 6.6±0.5 to 9.5±0.5. Almost no metabolites are observed in urine before 15 mins. Thus, the 15-min interval is considered too short for absorption upon ingestion [15]. Nevertheless, the appropriate interval remains unknown, and a 30-min interval may be too short as well [16].

### Limitations

One limit of this study is that the OFC were not double-blinded [17], which is standard. However, the subjects in this study were infants, many of which generally do not require double-blind OFC. The evaluation of subjective symptoms is one reason to conduct a double-blind OFC. However, mild subjective symptoms were not used as positive criteria in this study. Thus, they are expected to have minimal effect on the outcomes.

Further, our study design was a case-control study, not a randomized controlled study. Nevertheless, little difference was seen in the background between methods. Only immediate clinical history differed in the 30-min interval group, although the target background was not identical.

A significant difference in the clinical history of immediate-type symptoms was observed between the two groups. If the characteristics of the study population differ, it is hard to simply compare the results of OFC. Thus, the risk for subjects in the 30-min interval group may increase with immediate clinical history. However, the risk is unlikely to decrease. Therefore, it is not expected to affect the results significantly. Additionally, even after adjusting for the impact of immediate symptoms in multivariate analysis, the relative frequency of cardiovascular symptoms and adrenaline usage was significantly higher in the 15-min interval group than in the 30-min interval group. Thus, the 30-min interval method may avoid increased risks of cardiovascular symptoms and adrenaline administration. These could potentially account for less cardiovascular reactions and less adrenaline requirement in the 30-min group, as older children tend to grow out of wheat allergy regardless of the severity of previous reactions. However, strict examination in a randomized, controlled study is needed.

## Conclusions

The OFC method was changed from 15-min intervals to 30-min intervals in order to analyze various factors. Changing to a 30-min interval method did not increase the rate of symptom induction and treatment requirement. Furthermore, the 30-min interval method may avoid cardiac symptoms and the need for adrenaline administration.

The 30-min interval method was simpler than the 15-min interval alternative, resulting in greater safety with regard to symptom manifestation and more efficient observation. In future, safer and more efficient ways to standardize the OFC are needed.

## Supporting Information

S1 Table Characteristics of patients who required adrenaline. (DOC)
