# Correction: Do Longer Intervals between Challenges Reduce the Risk of Adverse Reactions in Oral Wheat Challenges?

**DOI:** 10.1371/journal.pone.0145567

**Published:** 2015-12-18

**Authors:** Noriyuki Yanagida, Takanori Imai, Sakura Sato, Motohiro Ebisawa

There are errors in Table 1. Please see the corrected Table 1 here.

**Table 1:** Grading of symptoms.

	1 (mild)	2 (moderate)	3 (severe)
Skin	Localized urticarial or exanthema or wheal or pruritis	Generalized urticarial or exanthema or wheal or pruritis	-
	Swollen eyelid or lip	Swollen face	-
Gastrointestinal tract	Pruritus of the throat or oral cavity	Throat pain	-
	Mild abdominal pain	Moderate abdominal pain	Cramps
	Nausea, emesis, diarrhea	Recurrent emesis or diarrhea	Continuous emesis, loss of bowel control
Respiratory tract	Intermittent cough, nasal congestion, sneezing, rhinorrhea	Repetitive cough	Persistent cough, hoarseness, “barky” cough
	-	Chest tightness, mild wheezing	Apparent wheezing, dyspnea, cyanosis, saturation <92%, swallowing or speaking difficulties, throat tightness, respiratory arrest
Cardiovascular	-	Pale face, mild hypotension, tachycardia (increase >15 beats/min)	Hypotension, dysrhythmia, severe bradycardia, cardiac arrest
Neurological	Change in activity level, tiredness	“Light-headedness,” feeling of “pending doom,” somnolence	Confusion, loss of consciousness, incontinence

The severity score should be based on the organ system most affected.

Hypotension was defined as systolic blood pressure of <70 mmHg (ages, 1 month to 1 year), <(70 mmHg + [2 × age]) (ages, 1–10 years), and <90 mmHg (>11 years).

Mild hypotension was defined as systolic blood pressure of <80 mmHg (ages, 1 month to 1 year), <(80 mmHg + [2 × age]) (ages, 1–10 years), and <100 mmHg (>11 years).

Total severity scores were defined as the grade of cardiovascular symptoms + the grade of respiratory symptoms + the maximum grade of other symptoms.

## References

[1] Yanagida N, Imai T, Sato S, Ebisawa M (2015) Do Longer Intervals between Challenges Reduce the Risk of Adverse Reactions in Oral Wheat Challenges? PLoS ONE 10(12): e0143717 doi:10.1371/journal.pone.0143717 26624006 10.1371/journal.pone.0143717 PMC4666606

