# Supplementary material for: Do Longer Intervals between Challenges Reduce the Risk of Adverse Reactions in Oral Wheat Challenges?
Source: PLoS One. 2015 Dec 1;10(12):e0143717. doi: 10.1371/journal.pone.0143717 (PMC4666606; doi:10.1371/journal.pone.0143717)
Supplement: S1 Table — (DOC) [file pone.0143717.s001.doc]

**S 1 Table**: Characteristics of patients who required adrenaline

| Case | Method | Age (years) | Gender | Wheat-specific IgE (kU_A_/L) | Elicting dose of wheat protein (mg) | Time of symptom development (min) | Symptoms |
| --- | --- | --- | --- | --- | --- | --- | --- |
| 1 | 15 min interval | 1.2 | Female | 49.3 | 156 | 30 (Resp) | Skin, Resp, Mucosal, Shock |
| 2 | 15 min interval | 1.7 | Male | 5.67 | 312 | 30 (Resp) | Skin, Resp, Shock |
| 3 | 15 min interval | 2.8 | Male | 4.95 | 312 | 40 (Resp) | Skin, Resp |
| 4 | 15 min interval | 2.5 | Male | 10.6 | 598 | 55 (Skin) | Skin, Resp |
| 5 | 15 min interval | 1.9 | Male | 4.25 | 806 | 60 (Resp) | Skin, Resp, Shock |
| 6 | 15 min interval | 5.2 | Male | 19.4 | 624 | 48 (Resp) | Skin, Resp, GI, Shock |
| 7 | 15 min interval | 5.9 | Female | > 100 | 624 | 50 (Resp) | Skin, Resp |
| 8 | 15 min interval | 1.7 | Male | 30 | 130 | 90 (Resp) | Skin, Resp |
| 9 | 15 min interval | 6.7 | Female | 44.6 | 130 | 65 (Resp) | Skin, Resp, Mucosal |
| 10 | 30 min interval | 1.8 | Female | 9.22 | 624 | 45 (Resp) | Skin, Resp |
| 11 | 30 min interval | 5.8 | Male | 13.8 | 624 | 30 (Resp) | Skin, Resp |
| 12 | 30 min interval | 7.9 | Female | > 100 | 130 | 60 (GI) | Skin, Resp, GI |

GI, Gastrointestinal; Resp, Respiratory
